# The plastid genome of a spice plants *Cinnamomum glanduliferum* in Tibet (Lauraceae)

**DOI:** 10.1080/23802359.2019.1671249

**Published:** 2019-09-27

**Authors:** Guanfei Zhao, Jie Yang, Xilong Wang, Yu Song, Rongjie Zhu

**Affiliations:** a Institute of Vegetable Sciences, Tibet Academy of Agricultural and Animal Husbandry Sciences, Tibet, China;; b Tibet Plateau Institute of Biology, Tibet, China;; c Center for Integrative Conservation, Xishuangbanna Tropical Botanical Garden, Chinese Academy of Sciences, Yunnan, China

**Keywords:** Cinnamomum, chloroplast, phylogenetic analysis

## Abstract

*Cinnamomum glanduliferum* (Wall) Meissn is a commercially important timber tree and wild spice plants of the genus *Cinnamomum* Trew in the family Lauraceae. To determine its phylogenetic location with respect to the other *Cinnamomum* species, the complete plastid genome of *C. glanduliferum* was sequenced. The whole plastome is 152,715 bp in length, consisting of a pair of inverted repeat (IR) regions of 20,114 bp, one large single copy (LSC) region of 93,617 bp, and one small single copy (SSC) region of 18,870 bp. The overall GC content of the whole plastome is 39.1%. Further, maximum likelihood phylogenetic analyse was conducted using 13 complete plastomes of the Lauraceae, which support close relationship between *C. glanduliferum* and *C. bodinieri.*

*Cinnamomum glanduliferum* (Wall) Meissn, a large-sized evergreen tree species, is widely distributed in Guizhou, Sichuan, Tibet, and Yunnan of SW China and Bhutan, Nepal, North India, and North Myanmar (http://foc.iplant.cn/). The essential oil isolated from the bark and leaf of *C*. *glanduliferum* are rich in eucalyptol (Azab et al. 2017; Taha and Eldahshan 2017) and thus represent important wild woody aromatic plants in the genus *Cinnamomum* Trew (Kumar and Kumari 2019). For a better understanding of the relationships of *C*. *glanduliferum* and other *Cinnamomum* species, it is necessary to reconstruct a phylogenetic tree based on high-throughput sequencing approaches.

About three gram dry leaves of *C*. *glanduliferum* in Chayu County (Tibet, China; Long. 96.772239 E, Lat. 28.723217 N, 1906 m) were collected for DNA extraction (Doyle and Dickson 1987). The voucher was deposited at the Biodiversity Research Group of Xishuangbanna Tropical Botanical Garden (Accession Number: XTBG-BRG-SY36414). The whole plastid genome was sequenced following Yang et al. (2014), and their nine universal primer pairs were used to perform long-range PCR for next-generation sequencing. The contigs were aligned using the publicly available plastid genome of *C*. *chago* (Accession number LAU00078) (Chen et al. 2019) and annotated in Geneious 4.8.

The plastome of *C*. *glanduliferum* (LAU00111), with a length of 152,715, was 45 bp and 4 bp smaller than that of *C*. *parthenoxylon* (152,760 bp, MH050971) and *C. bodinieri* (152,719 bp, MH394415). It was also 40 and 15 bp larger than that of *C. micranthum* (152,675 bp, KT833081) and *C*. *kanehirae* (152,700 bp, KR014245). It composed of a large single copy (LSC) region of 93,617 bp, a small single copy (SSC) region of 18,870 bp, and a pair of inverted repeat (IR) regions of 20,114 bp. The overall G + C content was 39.1% (LSC, 37.9%; SSC, 33.8%; IR, 44.4%).

Furthermore, based on 15 published plastid genome sequences, we reconstructed a phylogenetic tree (Figure 1) to confirm the evolutionary relationship between *C*. *glanduliferum* and other species with published plastomes in *Cinnamomum*, with *Laurus* species as outgroup (Song et al. 2017). Maximum likelihood (ML) phylogenetic analyses were performed base on K3Pu + F + I model in the iqtree version 1.6.7 program with 1000 bootstrap replicates (Nguyen et al. 2015). The ML phylogenetic tree with 41% to 100% bootstrap values at each node supported that *Cinnamomum* species grouped into two clades, and that *C*. *glanduliferum* and *C. bodinieri* were located in the same clade.

**Figure 1.**
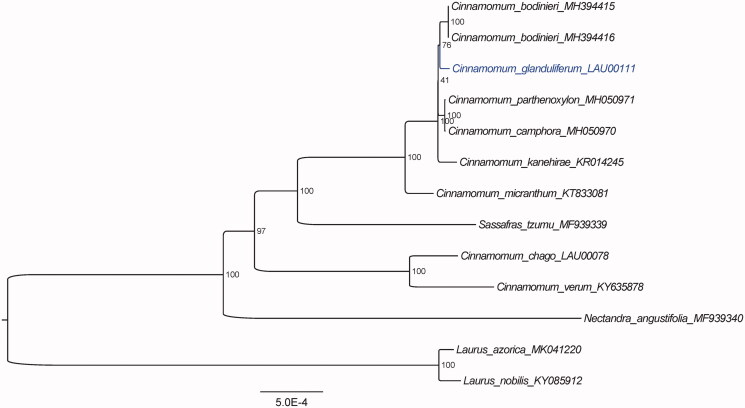
The ML phylogenetic tree for *C*. *glanduliferum* based on other 12 species (8 in *Cinnamomum*, 1 in *Nectandra*, 1 in *Sassafras* and 2 in *Laurus*) plastid genomes.

## Data Availability

The plastome data of the *C. glanduliferum* will be submitted to Lauraceae Chloroplast Genome Database (https://lcgdb.wordpress.com). Accession numbers are LAU00111.
